# 

**Published:** 1900-06-16

**Authors:** 


**BIRTH.**

Young.—On June 7th, at 45, Stamford Hill, the wife of John Young, M.D., B.S., of a son. (2,173)

### NOTICE TO CORRESPONDENTS.

All MS., Letters, Books for Review, and other matters intended for the Editor should be addressed The Editor, "The Hospital," The Hospital Building, 28 & 29, Southampton Street, Strand, W.C.

The Editor cannot undertake to return rejected MS., even when accompanied by stamped directed envelope.

**Notice to Advertisers and Subscribers.**

All Advertisements, Orders for Copies of The Hospital, and Business Communications should be addressed to **THE MANAGER (Not to the Editor)**, The Hospital, The Hospital Building, 28 & 29, Southampton Street, Strand, London, W.C.

The Cost of Subscription, post free, is as follows:—Per week, 2½d.; per quarter, 2s. 8d.; per half-year, 5s. 3d.; per annum, 10s. 6d. Foreign:—Per annum, 15s. 2d., payable, in all cases, in advance.

**NOTICE.—Vol. XXVII. of "The Hospital" (October, 1899, to April, 1900), in handsome cloth binding, is now on sale at the Office of this Journal, price 7s. 6d. Cases for binding the Numbers contained in this Volume 1s. 6d. Index to Vol. XXVII., 2½d., post free.**

**he Monthly Part for June is now ready. Price 6d., by post 8d.**

### BOOKS RECEIVED.

Adlard and Son.

"Notes on Modern Asylum Construction." By R. H. Steen, M.D.

John Bale, Sons, and Danielsson,

"The Early Treatment of Appendicitis." By Donald W. C. Hood, M.D., F.R.C.P.

C. Tinling and Co.

"Report on the Health of the City of Liverpool, 1899." By E. W. Hope, M.D., D.Sc., Medical Officer of Health.

T. Fisher Unwin.

"The Century Invalid Cookery Book." By Mary A. Boland. Edited by Mrs. Humphry ("Madge" of Truth).

Baillière, Tindall, and Cox.

"Appendicitis." By A. H. Tubby, M.S.Lond., F.R.C.S.

"The Bacteriology of Every Day Practice." By J. O. Symes, M.D. (State Medicine), Lond.

### SCRAPS AND GLEANINGS.

During 1899 the sum of £174 odd was realized by means of the annual house to house collection in aid of the funds of the Victoria Hospital for Sick Children, Hull.

The number of in-patients treated during 1899 at the Wolverhampton and District Hospital for Women was 203; out-patients 782. The income had been £991, and the expenditure £1,073.

The report for the year 1899 of the Ladies' Council of the Manchester Children's Hospital shows that there are now 15 cots bearing the names of the districts which have contributed the £50 necessary for that to be done. The board were satisfied that they were now in a position to establish a "ladies" ward, at an estimated cost of £1,500.

